# Annotations

**Published:** 1907-03-02

**Authors:** 


### ANNOTATIONS.

### The Sanitary Evolution of London.

Mr. Henry Jephson, a member of the London County Council, has recently published a book under this title, and in its pages may be found many facts of great social and economic interest and importance. Proclamation has been made on high authority that worthy service to the community may be rendered in the municipal not less than in the Parliamentary sphere, and there is abundant evidence in Mr. Jephson's book in support of this proposition. Taking the questions which especially appeal to the professional interests of our readers, we find, first, figures to show that within fifty years the death-rate in London has decreased from 23.38 to 17.1 per 1,000. This improvement is attributed principally to developments in the perfecting of the sanitary arrangements. The sanitary system includes nearly 90 miles of great intercepting and outfall sewers, 176 miles of main sewers, 26 miles of relief sewers, and more than 2,600 miles of local sewers. The amount of sewage dealt with per annum is said to equal a lake 11 1-5 feet deep and having an area of 44 square miles. Another piece of official machinery by which the health of the community is safeguarded is the inspection of food. In this respect the figures show that at Smithfield, in 1905, out of 415,000 tons of meat received, 2,128 tons were condemned as diseased or unsound; and at Billingsgate, 674 tons of the 211,600 of fish received, as well as 173 tons of tinned meat, were rejected on inspection and were ordered to be destroyed. It is calculated that the cost of disease in the Metropolis equals half a million sterling per annum, and this does not include the loss to the individual in wages or to the family by the incapacity of the breadwinner. Emphasis is laid on the necessity of improvement in domestic sanitary accommodation in many directions. There are even yet 304,874 persons who live in one-room houses, and 701,203 in two-room houses, and the death-rate under such conditions is proved to be abnormally high.

### The Royal Society of Medicine.

The movement for the amalgamation of the metropolitan medical societies has now begun to take definite shape, and at a recent meeting of the members of those societies which have officially intimated their adhesion to the scheme it was decided that the new organisation should be known as "The Royal Society of Medicine." We repeat our opinion that gratitude is due from the profession to those who have worked steadily and energetically to bring the amalgamation scheme to a successful issue. An organisation which will bring into close association, and for their mutual benefit, those who are working in the various departments of medicine is specially needed when, as at present, there is an increasing tendency to special practice. This tendency is inevitable, and to quarrel with it is useless. It arises from the very nature of things. But care can be taken to safeguard its developments and to prevent the dangers which are carried in its train. The amalgamation scheme will, we anticipate, be active in both these directions, and for this reason alone, and apart from others, it seems to us to deserve the support and goodwill of all interested in the progress of the science and art of medicine. Unfortunately, some of the more important societies have declined formally and officially to support it. But this by no means implies that all the members of these societies will stand aloof. On the contrary, many of these will doubtless join the new society, and it seems desirable that this fact shall be recognised at the earliest possible date. Though there may be official indifference, there will be many personal adhesions when the new organisation appeals directly to the profession.

### The Registrar-General's Report.

The sixty-eighth annual report of the Registrar-General deals with the births, deaths, and marriages which took place in England and Wales in 1905. It is, of course, a document of much interest and of high economic and national importance. In this brief reference to it we purpose merely to quote some few figures which bear upon subjects that have often been discussed in our columns. First, with regard to the birth-rate, it must be noted that the figures reach the lowest level that has been recorded since civil registration was established. The decline has been almost continuous since 1876, and the serious aspect of the question receives further emphasis from the fact that, with the single exception of France, the fertility of married women in England and Wales is now less than that of any European country. The conclusion is reached that, after allowing for a decrease in the proportion of married women of conceptive ages, and for a diminution in the number of illegitimate births, some 73 per cent. of the decline is due to the "deliberate restriction of child-bearing on the part of the people themselves." To the grave significance of such facts as are now placed on official record we have more than once directed attention. Turning next to the record of deaths, the report presents a more encouraging picture. The total number of deaths registered during the year was 520,031, or in the proportion of 15.2 per 1,000 persons living. This is the lowest rate on record, and is 8.2 per cent. below the average of the previous quinquennium. Regarding individual causes of death, tuberculosis was during the year responsible for 55,759 deaths, being fewer by 7,870 than the average annual number in the previous decennium. Under cancer, on the other hand, the numbers are 30,221, or more by 2,209 than the average number in the previous ten years. One other fact may be noted. It is that no death from hydrophobia has been recorded since 1902—an excellent illustration of the preventive treatment of disase. As a contrast, it is notworthy that in the official returns for London and seventy-five other great towns for the week ending February 9, 1907, no fewer than 14 infants were suffocated in bed—a cause of death that, as we have often pointed out, could be almost absolutely prevented by the adoption of common-sense precautions.

